# Evaluating the Skin Interactions and Permeation of Alginate/Fucoidan Hydrogels *Per Se* and Associated with Different Essential Oils

**DOI:** 10.3390/pharmaceutics15010190

**Published:** 2023-01-05

**Authors:** Ana Isabel Barbosa, Sofia A. Costa Lima, Ibraheem Yousef, Salette Reis

**Affiliations:** 1 LAQV, REQUIMTE, Departamento de Ciências Químicas, Faculdade de Farmácia, Universidade do Porto, Rua de Jorge Viterbo Ferreira, 228, 4050-313 Porto, Portugal; 2 LAQV, REQUIMTE, Departamento de Química, Instituto de Ciências Biomédicas de Abel Salazar, Universidade do Porto, Rua de Jorge Viterbo Ferreira, 228, 4050-313 Porto, Portugal; 3 ALBA Synchrotron, Carrer de la Llum 2-26, Cerdanyola del Vallès, 08290 Barcelona, Spain

**Keywords:** cutaneous delivery, marine polysaccharides, penetration enhancers, synchrotron-based Fourier transform infrared microspectroscopy, stratum corneum layer

## Abstract

Marine polysaccharides are recognized for their biological properties and their application in the drug delivery field, favoring hydrogel-forming capacities for cutaneous application towards several dermatological conditions. Essential oils have been widely used in skin, not only for their remarkable biological properties, but also for their capacity to enhance permeation through the skin layers and to confer a pleasant scent to the formulation. In this study, menthol, L-linalool, bergamot oil, and β-pinene were incorporated in alginate/fucoidan hydrogels to evaluate their skin permeation enhancement profile and assess their influence on the skin organization. The combinations of different essential oils with the marine-based fucoidan/alginate hydrogel matrix were characterized, resulting in formulations with pseudoplastic rheological properties favorable for a uniform application in the skin. The ex vivo Franz diffusion permeation assays revealed that calcein loaded in bergamot-alginate/fucoidan hydrogel permeated more than 15 mg out of the initial 75 mg than when in linalool-alginate/fucoidan, alginate/fucoidan or hydrogel without any incorporated oil. Skin calcein retention for menthol- and pinene-alginate/fucoidan hydrogels was 15% higher than in the other conditions. Infrared micro-spectroscopic analysis through synchrotron-based Fourier Transform Infrared Microspectroscopy evidenced a symmetric shift in CH_3_ groups towards higher wavenumber, indicating lipids’ fluidization and less lateral packing, characterized by a band at 1468 cm^−1^, with the bergamot-alginate/fucoidan, which contributes to enhancing skin permeation. The study highlights the effect of the composition in the design of formulations for topical or transdermal delivery systems.

## 1. Introduction

Marine polysaccharides are often in the spotlight due to their interesting biological properties, their high bioavailability and biocompatibility, and their potential application in the field of drug delivery [1]. Divided according to their pigments (green algae, *Chlorophyceae*; red algae, *Rhodophyceae*; and brown algae, *Phaeophyceae*), algae are considered one of the richest polysaccharide suppliers applied in different therapeutic areas [2,3], with a clear research focus on skin health [3] through the areas of wound dressing, tissue engineering [4,5], and dermocosmetic.

Alginate and fucoidan polymers are found in brown algae and have been reported in several nanotherapeutic studies [6,7], as their effective extraction remarkably improved their application in the drug delivery field [8]. In this work, alginate/fucoidan hydrogels were prepared to study their potential application as a skin delivery system. Alginate is composed of (1,4)-linked β-d-mannuronic and α-l-guluronic monomers, rearranged in various proportions depending on the extraction source [7], with effects on its hydrogel strength, transmittance, swelling, and viscoelastic properties [9,10]. Due to its capacity to incorporate large amounts of bioactive molecules, alginate has been extensively used in the design of several delivery systems [11], particularly in hydrogel production [12,13]. The main reported alginate properties and advantages are related to its biocompatibility, biodegradability, cell affinity, strong bioadhesion, high capacity to absorb water, inert nature and non-toxicity, ease of gelation and regeneration, and capacity to activate macrophages [14,15]. Fucoidan is essentially composed of L-fucose and sulfate ester groups, presenting different biological activities dependent on its source and its molecular weight, type of sugar content, sulfation degree, molecular structure, harvesting, and extraction conditions [16]. The most reported properties are antitumor [17,18,19,20,21], antiviral [22,23], anti-inflammatory [24,25,26,27,28,29], and anticoagulant [26,30,31,32,33], but also skin management potential [34,35,36,37]. Pozharitskaya and colleagues evidenced the cutaneous application of fucoidan with pharmacokinetic studies in rats, which indicated fucoidan’s ability to cross the skin barrier and accumulate in the striated muscles [38]. In the present work, the aim is to combine fucoidan’s topical pharmacokinetic potential with the current knowledge and potentialities of using alginate in wound healing and cutaneous drug delivery [39,40] to design a hydrogel for skin delivery.

Taking advantage of the aforementioned algal polysaccharides’ properties, in particular, their individual anti-inflammatory and hydrogel-forming capacities, could be interesting to develop delivery systems for cutaneous application. Hydrogels have been described as desirable drug delivery systems when compared to other topically applied galenic forms, especially due to their higher water content, with associated cooling and hydration effects, reduced transepidermal water loss, longer drug absorption, and high skin biocompatibility [41,42]. As the largest organ in the human body, the skin is a natural barrier that protects the organism against undesirable external aggression, but it is also the major hurdle to overcome when planning drug administration through this route. Among the well-known skin layers, the stratum corneum (SC) represents challenging horizontally packed layers of dead keratinocytes (corneocytes) embedded in a lipid matrix (commonly associated with a “brick and mortar” structure) with remarkable protein complexity, adaptation, and the self-maintenance capacity of the skin barrier function [43]. Any perturbance of this well-orchestrated balance can represent a strategy to perform an effective drug delivery through the skin by enhancing the permeation of active compounds through this desirable route [44]. Skin penetration can be enhanced by interaction with lipids, either by fluidizing or disorganizing lipids, and even extracting them; by interacting with proteins; and by increasing drug solubility and partitioning in the skin [45,46,47]. These phenomena are particularly achievable with the use of chemical penetration enhancers, such as terpenes, terpenoids, and essential oils [48]. Since the isolation of essential oils and the study of their volatile compounds, several pharmaceutical applications were reported [49,50], including their use as skin penetration enhancers for transdermal drug delivery [51]. Representing a good alternative to synthetic skin penetration enhancers, essential oils are cheaper and safer due to their higher clearance of the skin. After cutaneous application, components of essential oils are rapidly metabolized, resulting in non-accumulation and rapid excretion, though more clinical trials are needed to confirm the real safety of these plant-based products’ application in humans [52]. Essential oils can penetrate through the SC and enhance the cutaneous entry of both lipophilic and hydrophilic drugs, using mechanisms of fluidization and disintegration of the intercellular robust lipid structure of corneocytes in SC, by interacting with and modifying the conformation of intercellular proteins and increasing the drug partitioning [53]. Monoterpenes and sesquiterpenes correspond to 25% of the terpene fractions in essential oils [54]. Terpenes are also known for their ability to permeate the skin’s SC, as the smaller terpenes are the ones that permeate better [55,56]. In this context, menthol, L-linalool, bergamot oil, and b-pinene were selected for incorporation in alginate/fucoidan hydrogels to address their effect as skin permeation enhancers.

One of the most widely studied terpenes in the field of skin penetration enhancement is menthol [57]. Menthol is a cyclic monoterpene that was found to disrupt the intercellular lipids of SC, revealing changes in infrared spectra and X-ray diffraction patterns of SC, and consequently enhancing the skin permeation of the indomethacin by higher concentration and diffusion rate in the skin [58]. Recent studies show that menthol can be incorporated in the matrix of nanoparticles [59] and hydrogels [60] to work as skin permeation enhancers, and this effect is more evident in a concentration-dependent manner [61]. L-linalool is an acyclic terpene alcohol extracted from aromatic plants [62]. Linalool was found to improve the percutaneous absorption of propranolol [57] and was tested among other terpenes to improve the skin permeation active agents from tea (catechins and theophylline), showing one of the highest sets of results for flux, skin deposition, and enhancement ratios [63]. Linalool and linalyl acetate were comparatively studied due to their similar scent and anti-inflammatory properties [64]. In fact, some authors stated the hypothesis that linalool and linalyl acetate can be formulated in the same vehicle for the useful treatment of inflammation scenarios [65]. Bergamot oil is a commercially available essential oil extracted from *Citrus bergamia*, and it is mainly composed of limonene, linalyl acetate, and linalool [66]. A study disclosed the antinociceptive effect of bergamot oil and linalool after the intra-plantar combined injection of bergamot oil and capsaicin in mouse hind paw [67]. Associating the proven skin permeation enhancement effect of linalool and linalyl acetate, as well as their anti-inflammatory properties, the use of bergamot oil can be a good strategy to treat inflammation and overcome the skin barrier. Another minor component of bergamot oil with potential application in cutaneous penetration enhancement is b-pinene, which is a bicyclic monoterpene mostly found in pine essential oils, with interesting pharmacological effects, such as antioxidant, anti-inflammatory, and analgesic effects [68]. A comparative study performing in vitro human skin permeation of monoterpenes has shown that the apparent permeability coefficients of β-pinene were four times higher than other terpenes (e.g., β-myrcene, limonene, and α-pinene) [69]. This study aimed to unravel and evaluate the mechanism of the inherent permeation profile of marine polymeric hydrogels *per se* and associated with menthol, linalool, bergamot oil, and pinene, which belong to the class of skin penetration enhancers. This study involved the hydrogel’s physicochemical characterization and ex vivo skin permeation assays of calcein, a model compound that is commonly used to validate the effect of differently skin-targeted platforms of the proposed model [70,71,72]. To study skin organization regarding lipid and protein structures, infrared spectroscopic analysis through synchrotron-based Fourier Transform Infrared Microspectroscopy (SR-FTIRM) was performed for the SC layer, the first hurdle to overcome in order to achieve cutaneous delivery.

## 2. Materials and Methods

### 2.1. Materials

Fucoidan extracted from *Fucus vesiculosus* (92.9% fucoidan phytonutrient content, high molecular weight, code 5619002900, batch FVF2016507) was a kind gift from Kraeber & Co. GMBH (Ellerbek, Germany). Sodium alginate (from *Lessonia nigrescens*, code 177775000, lot A0376873, sulfated ash 34.16% (on dried substance)) was purchased from ACROS OrganicsTM (Thermo Fisher Scientific, Waltham, MA, USA). (−)-Menthol was acquired from Fluka (Buchs, Switzerland). Bergamot oil was purchased from Pranarôm (Ghislenghien, Belgium). L-Linalool, (−)-β-pinene, calcein, 4-(2-hydroxyethyl)piperazine-1-ethanesulfonic acid (HEPES) hemisodium salt, and trypsin (from the porcine pancreas) were acquired from Sigma-Aldrich (St. Louis, MO, USA). The porcine ears were purchased at a local slaughter (Porto, Portugal). Double-deionized water was provided by an ultra-pure water system (Arium Pro, Sartorius AG, Gottingen, Germany). The reagents were weighted in a digital analytical balance Kern ACJ/ACS 80-4 (Kern & Sohn; Balingen, Germany). All other reagents were analytical grade and were used without any further purification.

### 2.2. Preparation of Hydrogels

For hydrogel preparation, and based on different percentage combinations of both polymers, 1% (*w*/*v*) sodium alginate and 2% (*w*/*v*) fucoidan were simultaneously dissolved in double-deionized water, as a way to obtain a higher amount of the sulfated polysaccharide in the matrix and to have a concentration of the carboxylated polysaccharide able to achieve gelation. After complete dissolution, 1% (*w*/*v*) of each essential oil was used to enrich the hydrogels, using 5 min vortex to facilitate its incorporation in the matrix. In calcein-loaded hydrogels, 1% (*w*/*w*) of calcein was also added to the hydrogel blend in the dissolution step. After each hydrogel preparation, the final pH was measured with a Crison pH meter GLP 22 with a Crison 52-02 tip (Crison; Barcelona, Spain).

### 2.3. Physicochemical Characterization of Hydrogels

#### 2.3.1. Morphological Analysis

The hydrogels were frozen overnight at −80 °C (Deep Freezer, GFL^®^, Burgwedel, Germany) and then lyophilized using a freeze drier (LyoQuest -85 plus v.407, Telstar^®^ Life Science Solutions, Terrassa, Spain) for 72 h at −80 °C under 0.40 mbar of pressure. The freeze-dried hydrogels were analyzed by Scanning Electron Microscopy (SEM) using a FEI Quanta 400 FEG ESEM/EDAX Pegasus X4M with an accelerating voltage of 10 kV. Hydrogels were fixed onto carbon-taped metal pins and coated with Au/Pd by sputtering for 45 s.

#### 2.3.2. Differential Scanning Calorimetry Analysis

Measurements were performed using a differential scanning calorimeter (DSC 200 F3 Maia Netzsch). Approximately 5–10 mg of all individual components and freeze-dried hydrogels were weighed in aluminum pans and sealed. An empty aluminum pan was used as a reference. Heating curves were recorded with a heating rate of 10 K/min from −40 °C to 300 °C and software provided by the DSC equipment (NETZSCH Proteus^®^ Software—Thermal Analysis—Version 6.1).

#### 2.3.3. Rheology Studies

The rheological properties of the prepared hydrogels were analyzed on a rheometer (Malvern Kinexus Lab+; Malvern Instruments; Worcestershire, UK) using four different methods. For viscosimetry, a shear rate table method (0.1 to 100.0 s^−1^, 10 samples per decade, 25 °C) was used. The thixotropy test followed a three-step shear rate method (1st phase: 0.1 s^−1^, 2 min; 2nd phase: 100.0 s^−1^, 30 s; 3rd phase: 0.1 s^−1^, 15 min, 25 °C). To determine the linear viscoelastic region, an amplitude sweep method was performed (0.1 to 100%, 10 samples per decade, 1.0 Hz, 25 °C). Finally, to address the temperature effect, a single frequency temperature ramp was used (initial temperature 20 °C, final temperature 40 °C, 2 °C/minute ramp, frequency 1 Hz). All analysis was conducted with a plate-plate configuration (geometry CP4/40 SR4321) with a 1 mm gap (Peltier Plate Cartridge). All experiments were performed in triplicate, and data were collected using the rSpace software^®^ (Kinexus 1.75: PSS0211-17).

### 2.4. Ex Vivo Skin Permeation Assays

The ex vivo skin permeation assays were performed using porcine ear skin as a barrier. The skin was obtained from a local butcher, immediately detached using a scalpel, cleaned, and stored in plastic bags at −20 °C. The assay was performed in a Franz cell assembly (9 mm unjacketed Franz Diffusion Cell with 5 mL receptor volume, O-ring joint, clear glass, clamp, and stir-bar; PermeGear, Inc., Hellertown, PA, USA). A volume of 250 µL of the hydrogels or free calcein corresponding to 75 µg was placed on top of the full skin. The receptor chamber was filled with HEPES buffer, pH 7.4, and was kept at 37 °C and stirred at 400 rpm during the assay. At the end of the assay, the amount of calcein in the donor and receptor compartments was determined by spectrofluorimetry (excitation and emission wavelengths at 495 nm and 517 nm, respectively) to overcome interferences from skin components released during the experiment. The amount of calcein retained in the skin was calculated considering the difference between the initial amount of calcein added to the donor compartment and the quantified amount at the end of the assay in the receptor compartment. The permeability results were expressed in the percentage of release and apparent permeability (*P*_app_). *P*_app_ was calculated using the following equation:

$$P\mathrm{app}\;\left(\mathrm{cm}/s\right)=\frac{\sum m_{a}}{A\times m_{d}\times t}$$

where the sum of *m_a_* is the mass of calcein permeated across the skin layer in the receptor chamber, *A* is the diffusion area between cells in the Franz diffusion system (0.64 cm^2^), *m_d_* is the initial mass of calcein in the donor chamber, and *t* is the time (24 h = 86,400 s).

### 2.5. Synchrotron-Based Fourier Transform Infrared Microspectroscopy

#### 2.5.1. Experimental Setup for SR-FTIRM Samples

Frozen porcine ear skin was cut as discs of 12 mm diameters, thawed at room temperature, placed in 24-well plates, and distributed considering the experimental groups: control (untreated), essential oils, hydrogel (without essential oils), and essential-oil-enriched hydrogels. Each disc was treated by applying 40 µL of each sample, enough to cover the area of the skin. All discs were incubated at room temperature for 24 h. After an incubation period, the excess of the sample was removed with a swab, and the skin was gently washed with a cotton swab impregnated with ultrapure water before further processing. The treated skin samples were frozen in liquid nitrogen before cutting in a cryostat in sections of 5 µm thickness and placed on CaF2 circular windows (Crystran Ltd., Poole, UK) for analysis under the synchrotron-based Fourier Transform Infrared Microspectroscopy (SR-FTIRM).

#### 2.5.2. Infrared Microspectroscopy at Synchrotron

The infrared microspectroscopy analysis was performed at the MIRAS beamline at the ALBA synchrotron (Cerdanyola del Vallès, Spain). The measurements were obtained using the 3000 Hyperion microscope coupled to a Vertex 70 spectrometer, equipped with a liquid nitrogen-cooled mercury cadmium telluride 50 m MCT-A detector. The spectra were collected using a Schwarzschild 36× magnification objective (0.52 NA) coupled to a 36× magnification condenser, with a single masking projected aperture size on the sample of 10 × 10 µm^2^ and step size of 5 × 5 µm^2^. Raster scan map measurements of a selected area on the skin were obtained in the 4000–900 cm^−1^ mid-infrared range at a spectral resolution of 4 cm^−1^, with 64 co-added scans per spectrum at room temperature in transmission mode. A background spectrum was collected every 15 measurements to eliminate the residual contamination (water vapor, CO_2_) of the room ambient. To extract spectral information, each spectrum was manually grouped considering the SC layer. Data treatment and statistical analysis were conducted as previously described using OPUS (Bruker, Billerica, MA, USA) and Unscrambler^®^ (CAMO software, Oslo, Norway) software [73,74].

### 2.6. Statistical Analysis

Statistical analysis was performed using GraphPad Prism Software (Version 6.01 for Windows; GraphPad Software Inc., San Diego, CA, USA). The One-Way ANOVA following Tukey’s multiple comparison test was performed to evaluate differences in pH values of all hydrogels, and also to compare free calcein and calcein-loaded hydrogels in the ex vivo permeation assay. Differences were considered significant at *p* < 0.05.

## 3. Results and Discussion

### 3.1. Characterization of Hydrogels

Addressing the skin pH should be a priority when designing skin formulations, and controlling its pH is a crucial and tunable step to accomplish higher skin penetration. For all hydrogels, the pH was determined in three independent batches. Alginate/fucoidan presented a pH value of 5.34 ± 0.18, and the inclusion of essential oils slightly lowers this value. Compared with alginate/fucoidan, menthol-alginate/fucoidan and pinene-alginate/fucoidan hydrogels present a similar pH value (5.20 ± 0.02 and 5.12 ± 0.01, respectively), but there is a statistical difference (** p < 0.01) obtained for linalool- and bergamot-alginate/fucoidan hydrogels with pH values below 5 (4.95 ± 0.02 and 4.96 ± 0.10, respectively). Skin is the first barrier that confers protection from external aggressions, and its microbiome and integrity are in a complex balance between several factors. The changes in skin pH can have pathogenetic consequences, leading to impaired skin barrier function and bacterial colonization [75]. The maintenance of an acidic pH is crucial for normal SC lipid organization and lipid metabolism [76]. The effective skin pH results from the interplay between the active compound, the formulation used in its delivery, and also the actual skin pH value [77]; hence, all these acidic hydrogel formulations might help re-establishing skin pH below 5, which is ideal for its native microbiome, with and without essential oils in its matrix [78].

**Figure 1:**
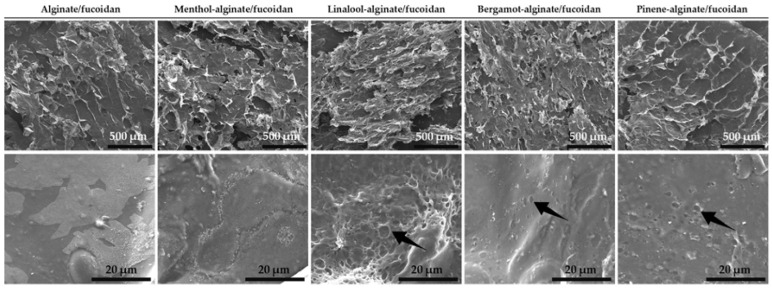
SEM micrographs of hydrogels (top scale bars: 500 µm; bottom scale bars: 20 µm).

The morphological analysis of all the designed hydrogels resulted in the scanning electron microscopy (SEM) micrographs shown in Figure 1. Considering the first set of images with a scale bar corresponding to 500 µm, the similarity in terms of macrostructure can be confirmed for all analyzed samples. The obtained branched scaffolds have also been described in previously freeze-dried formulations containing fucoidan and alginate [79]. Within the microstructure of the linalool-, bergamot-, and pinene-alginate/fucoidan hydrogels, observed with local amplification (Figure 1, second set of images, with a scale bar corresponding to 20 µm), it is possible to observe the presence of pores, evidenced with arrows. The essential oil droplets in the hydrogel matrix, upon freeze-drying, may result in these structures. The presence of pores might represent a good strategy to promote the controlled release of active substances from the hydrogel material, as well as serve as a hydrogel-incorporated platform to entrap hydrophobic compounds with good affinity to the oil phase [80].

**Figure 2:**
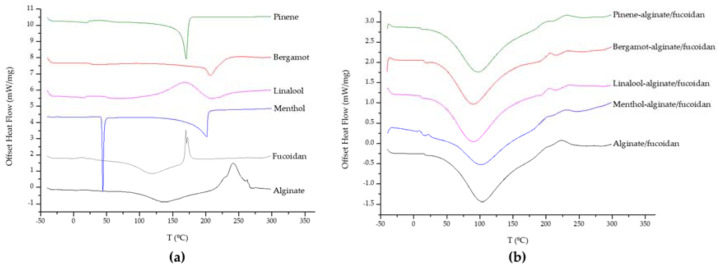
Offset thermograms of hydrogel components (**a**) and all hydrogels (**b**).

Also from SEM analysis, it was possible to observe crystalized menthol on the microstructure of menthol-alginate/fucoidan hydrogel. This was further evaluated by differential scanning calorimetry (DSC) analysis of all samples to determine the existence of phase transitions. The heat flow curves were obtained for all individual components used in the preparation of the hydrogels (Figure 2a). The alginate heat flow curve indicates some residual water evaporation after 100 °C, melting exothermic peaks between 225 and 240 °C, and degradation around 260 °C [81]. The residual water evaporation can also be found in fucoidan polymer, as well as a degradation-related exothermic peak around 170 °C [82]. Menthol exhibited a melting endothermic transition peak around 35–40 °C and another around 200 °C, which can be attributed to thermal oxidation [83]. Linalool, bergamot, and pinene oil curves showed endothermic peaks at about 205, 210, and 170 °C, respectively [84,85]. The thermal behavior of all hydrogels (Figure 2b) also revealed the evaporation of residual water present in the matrix, but the individual characteristic peaks from the essential oils were not so evident in the heat flow curves, suggesting a complex formation and a successful embedding of the essential oils in the hydrogel matrix, comparable with the alginate/fucoidan platform [85,86].

### 3.2. Hydrogels’ Rheological Analysis

**Figure 3:**
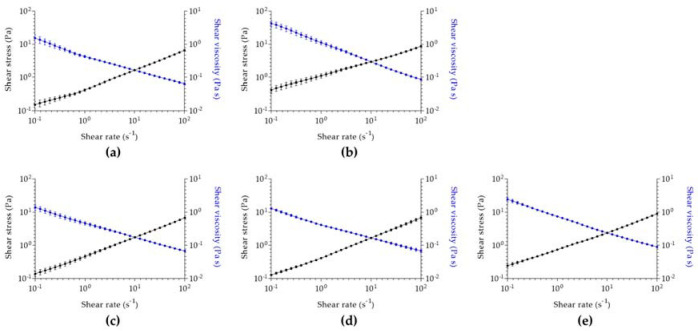
Viscosimetry analysis of (**a**) alginate/fucoidan, (**b**) menthol-alginate/fucoidan, (**c**) linalool-alginate/fucoidan, (**d**) bergamot-alginate/fucoidan, and (**e**) pinene-alginate/fucoidan. Data expressed as mean ± SD of n = 3 different hydrogel batches.

Aiming for a cutaneous application and to understand how the designed hydrogels will flow through the skin, a rheological analysis was performed for all samples. Regarding viscosimetry analysis (Figure 3), all hydrogels have similar initial viscosity, being higher in the case of menthol-alginate/fucoidan. Despite this difference, a shear thinning behavior is observed in all hydrogels. Since shear stress increases and shear viscosity decreases with increasing shear rate in all evaluated samples, the hydrogels are characterized by a pseudoplastic behavior. Pseudoplasticity indicates that a viscous formulation under static conditions will become less viscous after the application of shear stress, which will represent the motion of hydrogel application in the skin [87]. This property allows a higher spreadability of the formulation, enhancing uniform distribution in the skin [88] and contributing to a higher permeation of the active substances upon cutaneous application [87].

**Figure 4:**
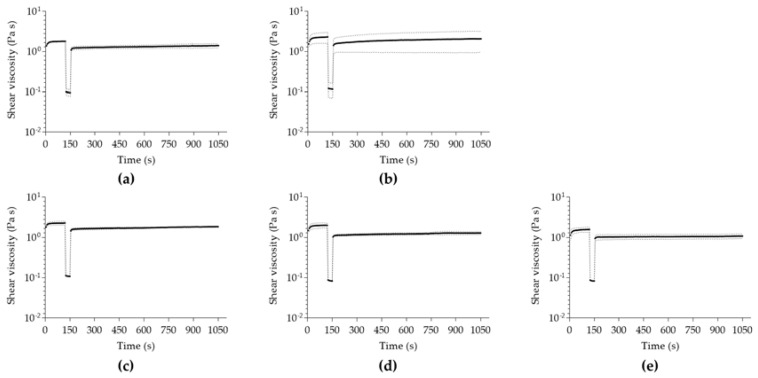
Thixotropy analysis of (**a**) alginate/fucoidan, (**b**) menthol-alginate/fucoidan, (**c**) linalool-alginate/fucoidan, (**d**) bergamot-alginate/fucoidan, and (**e**) pinene-alginate/fucoidan. Data expressed as mean ± SD of n = 3 different hydrogel batches.

In all analyzed samples, the initial viscosity (Figure 4) is not completely re-established during the rebuild time, after a high shear was applied to induce extreme stress conditions (Figure S1). This suggests a non-thixotropic profile of all hydrogels because the initial viscosity is higher than the one obtained by the recovered hydrogel matrix. The complex thixotropy of the essential-oil-enriched hydrogels might represent an interesting characteristic for the design of novel pharmaceutical formulations, particularly in skin formulations, determining the spreadability and interplay between the formulation and different skin layers [89] once the hydrogel structure is still maintained, even though with lower viscosity values.

All samples were also submitted to a temperature ramp set from 20 to 40 °C to evaluate their resistance by monitoring changes in elastic and viscous modulus. Analyzing the temperature ramp in all hydrogels (Figures S2 and S3), a slight viscosity reduction due to temperature increase is visible, which is a general effect in all tested formulations. There is great resistance to temperature increase in all hydrogels, except for menthol-alginate/fucoidan, which reflects a possible phase transition behavior around 34 °C, as evidenced by the menthol heat flow curve. Apart from the previous, the designed hydrogels might be stored within the set temperature range, and skin temperature might not be a crucial factor to change its initial properties [90]. These hydrogels’ capacity to maintain their characteristics in the set temperature range will be important for predicting the formulation behavior upon skin application, normally at 32 °C.

### 3.3. Hydrogels-Skin Interaction Studies

#### 3.3.1. Ex Vivo Skin Permeation

**Figure 5:**
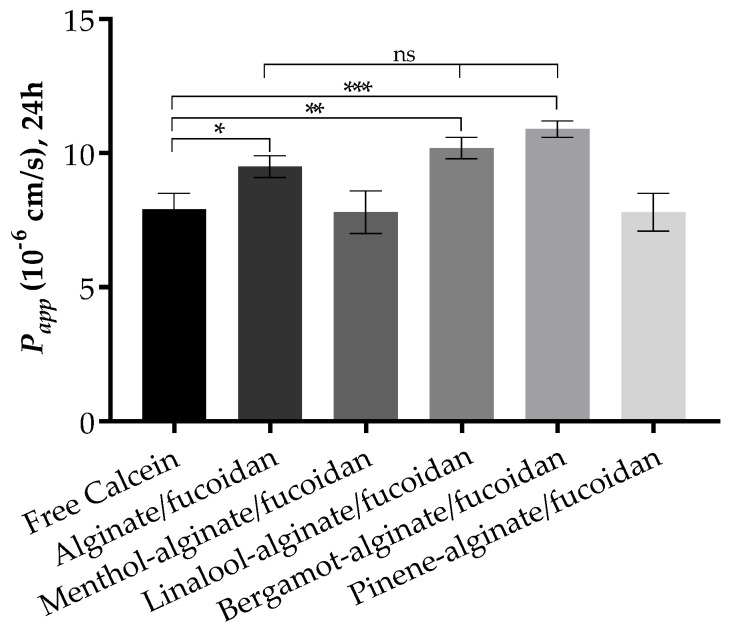
Apparent permeability at 24 h through pig ear skin for all tested sample conditions. Data expressed as mean ± standard deviation of n = 3 replicates. * *p* < 0.05; ** *p* < 0.01; *** *p* < 0.001.

The Franz cell diffusion assays were performed to evaluate the permeation of calcein from the designed hydrogels through full ex vivo pig ear skin. The pig ear is an interesting model to consider due to its similarity with human skin in terms of morphology and function [91,92], used without major ethical constraints. The use of some essential oils has been reported not only to achieve higher skin permeation—optimal for a transdermal delivery—but also to obtain higher retention in skin layers when a local effect is required [93,94]. The results reveal an increase in the permeation of calcein upon incorporation in some of the essential-oil-enriched alginate/fucoidan hydrogels at the end of 24 h assay, as expressed by apparent permeability data (Figure 5). The bergamot-alginate/fucoidan hydrogel permeates to a higher extent through the pig ear skin, followed by linalool-alginate/fucoidan and alginate/fucoidan hydrogels. Favoring the highest permeation enhancement, bergamot oil has also been studied for its antimicrobial effects on bacteria commonly involved in skin diseases [95]. In the cases of alginate/fucoidan, linalool-alginate/fucoidan, and bergamot-alginate/fucoidan, the Papp is statistically higher than free calcein. However, there is no statistical difference between these three samples, indicating that alginate/fucoidan hydrogel permeates the skin layers independently of the presence of these essential oils. Considering skin retention, menthol-alginate/fucoidan and pinene-alginate/fucoidan are the platforms that can retain up to 36% and 34%, respectively, of the initial amount of calcein, while the other platforms retain calcein up to 20% of the initial amount. Particularly in the case of menthol, this oil has been used to improve not only skin penetration, but also drug retention in skin appendages for a local effect [96]. The differential outcomes both in permeation and retention according to the essential oil used might help to design local or transdermal delivery of active compounds.

#### 3.3.2. Synchrotron-Based Fourier Transform Infrared Microspectroscopy of SC

Epidermal SC lipids are evenly distributed between free fatty acids, cholesterol, and ceramides, constituting a well-organized barrier to prevent water loss, microorganism infections, and general external aggressions [97]. This layer represents the first challenge to overcome to achieve cutaneous delivery. The SR-FTIRM analysis was explored as a high spatial resolution tool to understand the effect of essential oils as SC permeation enhancers alone, or in combination with the designed marine polymer-based hydrogel. In skin’s unique infrared fingerprint, it is possible to identify the long alkyl chains vibrations (2800–3000 cm^−1^), particularly asymmetric CH_2_ stretching (2920 cm^−1^) and symmetric CH_2_ stretching (2850 cm^−1^), whose changes elucidate the chain conformation, organization, and order of SC lipids [46,98]. Different phase transitions can be identified by the chain conformation and lateral packing of lipids, namely: orthorhombic phase, with all-trans aliphatic chains and rectangular crystalline lattice packing; hexagonal phase, with all-trans aliphatic tilted chains and less dense lattice packing; and liquid crystalline phase, governed by gauche isomerization and loss of laterally organized lattice packing [46]. Hence, skin penetration enhancers might fluidize lipids, promoting higher fluidity and flexibility of the alkyl chains, evidenced by higher frequencies and broader peak widths, and the opposite is evidenced by peaks at lower wavenumbers when a more ordered state takes place [99].

The analysis of significant changes regarding lipid stretching revealed that oils and essential-oil-enriched hydrogels alter lipid chain conformation towards lower fluidity, particularly evidenced by changes in CH_2_ symmetric and asymmetric stretching towards lower wavenumbers (Table 1). Besides these changes toward lower fluidity, bergamot-alginate/fucoidan also reveals a symmetric shift in CH_3_ groups towards higher wavenumber, indicating possible fluidization of the lipid matrix, thus explaining the higher permeation extent described in skin permeation studies.

The lipid lateral packing and integrity were studied through manifestations in lipid scissoring, addressing significant changes between 1480 and 1460 cm^−1^ [100]. A hexagonal packing of lipids is characterized by a band at 1468 cm^−1^, while the orthorhombic packing is identified by two components at 1472 cm^−1^ and 1464 cm^−1^ [76]. All essential oils and essential-oil-enriched hydrogels evidence the band at the 1468 cm^−1^ band, representing a hexagonal packing of lipids (Table S1). The band around 1454 cm^−1^ is related to the C–H bending in CH_3_ groups. Overall, samples manifest a band shift in this area when compared to untreated sample, corroborating the observed band shifts obtained in lipid CH_3_ stretching. However, it is important to point out the higher wavenumber for bergamot-alginate/fucoidan, which is consistent with the observed higher lipid stretching vibration wavenumbers. These changes in lateral packing suggest a higher fluidity and compromised barrier integrity after skin treatment with this hydrogel. This is also consistent with lipid stretching information, indicating that bergamot-alginate/fucoidan will diffuse better through the SC, increasing its permeation through the skin layers.

**Figure 6:**
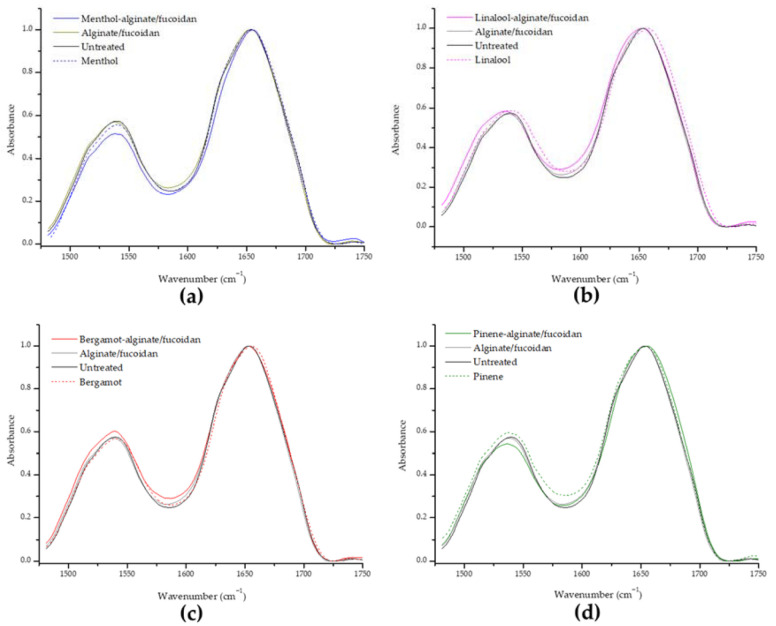
Evaluation of the amide I and amide II keratin band shifts in all essential oils and essential-oil-enriched hydrogels in comparison to the untreated sample, organized as menthol-alginate/fucoidan (**a**), linalool-alginate/fucoidan (**b**), bergamot-alginate/fucoidan (**c**), and pinene-alginate/fucoidan (**d**).

Protein alterations can be identified between the range of 1400 to 1800 cm^−1^, with major contributions from amide I (1600–1700 cm^−1^) and amide II (1480–1600 cm^−1^) bands. Keratin is the main protein in surface skin, and secondary structure and conformation can be monitored through changes in the amide I. Analyzing structural changes in proteins (Figure 6), no significant changes were found for amide I or amide II characteristic peaks of keratin, suggesting that the essential oils or hydrogel samples do not affect the protein component of SC.

SC keratin presents an α-helix secondary structure, and if this conformation is disturbed, changes in the amide I can be observed [74,101]. As previously mentioned, the denaturation of keratin can be a mechanism of penetration enhancement. Due to this ability to disrupt ordered SC lipid bilayers or force protein-mediated partition or denaturation in a concentration-dependent manner, skin penetration enhancers can be classified as cytotoxic, irritant, and allergenic [53]. The goal is to achieve penetration potency without compromising skin safety. In this context, all of the designed essential-oil-enriched hydrogels can be considered non-irritant because no structural changes take place.

In order to explain the mechanisms behind penetration enhancers, Brian Barry enounced the Lipid Protein Partition theory of skin penetration enhancement [102], on which this study was based. Skin permeation can occur through sweat pores and hair shafts, but mainly through intact skin SC, paving the path within the intercellular, intracellular, and transappendageal routes [47]. As such, compounds can interact with lipids, favoring their best affinity either to aqueous regions among polar head groups or among lipophilic regions of the alkyl chain bilayers. Corneocytes are the major components of SC, having keratin in their composition. As penetration enhancement can also occur by protein modifications, the partition theory considers the major alterations in lipid and protein structures, as well. Studies refer to the use of terpenes to fluidize and disorganize the SC, but other enhancer effects can be the promotion of lipid extraction, an increase of drug solubility and partition among lipidic bilayers, and even keratin disruption and detachment, causing defects in the normally tightly packed corneocytes [48]. Regarding synchrotron microspectroscopy analysis, the lipid structure of SC is not altered after treatment with the defined amounts of hydrogels or essential oils, except for the bergamot-alginate/fucoidan formulation. This is an interesting outcome when the aim is to fluidize the lipidic matrix to achieve higher penetration, but it could suggest that skin might be compromised or sensitized, as decreased lipid content or shorter lipid chains are common in skin diseases [103]. The absence of keratin alterations is also a positive indicator of the safe application of these marine-based polysaccharide combinations. Alginate/fucoidan with or without the essential oils can be applied as topical products without causing protein denaturation or disruption because keratin is responsible for maintaining the architecture and differentiation of epithelial cells, protecting the structural integrity of tissues, and promoting wound healing [104].

## 4. Conclusions

This study focused on the addition of 1% (*w*/*v*) of menthol, linalool, bergamot oil, and pinene to an alginate/fucoidan hydrogel matrix in order to study the permeation effects and skin interactions of the marine polymers *per se* or in combination with permeation enhancers. The pH values around 5 of the natural compounds-based hydrogels might help to balance the skin environment to optimal conditions in order to fight dysbiosis of inflamed skin, and with a rheological performance allowing a uniform spreadability of the formulation, promoting higher dermal permeation. Permeation studies in pig ear skin revealed that bergamot-alginate/fucoidan, linalool-alginate/fucoidan, and alginate/fucoidan improved full skin permeation of calcein when compared to the delivery of the free compound, while menthol- and pinene-alginate/fucoidan hydrogels favored calcein’s skin retention, optimal for a local delivery effect, with the controlled systemic distribution. The high skin permeation of calcein resulting from the application of alginate/fucoidan hydrogel also highlights its possible use as a platform for skin delivery, regardless of the addition of any penetration enhancer. SR-FTIRM data suggested that all essential oils and hydrogel formulations kept the SC lipid organization, only evidencing higher lipid fluidity and a less laterally packed lipid membrane after treatment with bergamot-alginate/fucoidan. This is consistent with the higher apparent permeability value obtained in the ex vivo pig skin Franz permeation result for bergamot-alginate/fucoidan, which can also suggest possible skin sensitization. As protein conformations are maintained in all hydrogels, these systems can also be considered non-irritant, a hypothesis that can be confirmed in the future with test guidelines on the safety of chemicals and mixtures following methods that closely mimic the biochemical and physiological properties of human skin. Further studies regarding the potential anti-inflammatory effect of the designed hydrogels and the absence of irritation and skin sensitization might help to reinforce the use of these formulations in future cutaneous delivery applications.

## Figures and Tables

**Table 1:** Evaluation of the lipid CH_2_ and CH_3_ stretching band shifts in all essential oils and essential-oil-enriched hydrogels.

	CH_2_ (cm^−1^)		CH_3_ (cm^−1^)	
	Symmetric	Asymmetric	Symmetric	Asymmetric
Menthol	2852	2922	2871	2958
Linalool	2852	2920	2871	2958
Bergamot	2850	2924	2873	2958
Pinene	2850	2918	2871	2958
Untreated	2852	2925	2871	2962
Alginate/fucoidan	2850	2922	2871	2958
Menthol-alginate/fucoidan	2850	2920	2871	2958
Linalool-alginate/fucoidan	2850	2922	2871	2956
Bergamot-alginate/fucoidan	2850	2920	2873	2960
Pinene-alginate/fucoidan	2852	2920	2873	2960

## Data Availability

Not applicable.

## Supplementary Materials

The following supporting information can be downloaded at: https://www.mdpi.com/article/10.3390/pharmaceutics15010190/s1, Table S1. Evaluation of lipid scissoring band shifts in all essential oils and essential oil-enriched hydrogels in comparison to the untreated sample. Figure S1. Analysis of (a) viscosity as a function of shear rate of alginate/fucoidan (black), menthol-alginate/fucoidan (blue), linalool-alginate/fucoidan (magenta), bergamot-alginate/fucoidan (red) and pinene-alginate/fucoidan (green). Calculated values of initial (minimum shear rate—0.1 s^−1^) and final (maximum shear rate—100 s^−1^) viscosity (b). Data expressed as mean ± SD of n = 3 different hydrogel batches. Figure S2. Resistance to deformation analysis from determination of linear viscoelastic region of (a) alginate/fucoidan, (b) menthol-alginate/fucoidan, (c) linalool-alginate/fucoidan, (d) bergamot-alginate/fucoidan and (e) pinene-alginate/fucoidan. Data expressed as mean ± SD of n = 3 different hydrogel batches. Figure S3. Resistance to temperature ramp from 20 to 40 °C of (a) alginate/fucoidan, (b) menthol-alginate/fucoidan, (c) linalool-alginate/fucoidan, (d) bergamot-alginate/fucoidan and (e) pinene-alginate/fucoidan. Data expressed as mean ± SD of n = 3 different hydrogel batches.
