# Supplementary material for: Evaluating the Skin Interactions and Permeation of Alginate/Fucoidan Hydrogels Per Se and Associated with Different Essential Oils
Source: Pharmaceutics. 2023 Jan 5;15(1):190. doi: 10.3390/pharmaceutics15010190 (PMC9861241; doi:10.3390/pharmaceutics15010190)
Supplement: Supplementary file 1 [file pharmaceutics-15-00190-s001.zip › pharmaceutics-2107727-supplementary.pdf]

pharmaceutics

Supplementary Material

Evaluating the Skin Interactions and Permeation of Alginate/Fucoidan Hydrogels *Per Se* and Associated with Different Essential Oils

Ana Isabel Barbosa [1], Sofia A. Costa Lima [2,*], Ibraheem Yousef [3] and Salette Reis [1]

[1] LAQV, REQUIMTE, Departamento de Ciências Químicas, Faculdade de Farmácia, Universidade do Porto, Rua de Jorge Viterbo Ferreira, 228, 4050-313 Porto, Portugal
[2] LAQV, REQUIMTE, Departamento de Química, Instituto de Ciências Biomédicas de Abel Salazar, Universidade do Porto, Rua de Jorge Viterbo Ferreira, 228, 4050-313 Porto, Portugal
[3] ALBA Synchrotron, Carrer de la Llum 2-26, Cerdanyola del Vallès, 08290 Barcelona, Spain
* Correspondence: slima@icbas.up.pt

Citation: Barbosa, A.I.; Lima, S.A.C.; Yousef, I.; Reis, S. Evaluating the Skin Interactions and Permeation of Alginate/Fucoidan Hydrogels *Per Se* and Associated with Different Essential Oils. *Pharmaceutics* **2023**, *15*, 190. https://doi.org/10.3390/pharmaceutics15010190

Academic Editor: Thierry Vandamme

Received: 3 December 2022
Revised: 27 December 2022
Accepted: 30 December 2022
Published: 5 January 2023

Copyright: © 2023 by the authors. Licensee MDPI, Basel, Switzerland. This article is an open access article distributed under the terms and conditions of the Creative Commons Attribution (CC BY) license (https://creativecommons.org/licenses/by/4.0/).

Table S1. Evaluation of lipid scissoring band shifts in all essential oils and essential oil-enriched hydrogels in comparison to the untreated sample.

	Scissoring	
Menthol	1454	1468
Linalool	1452	1468
Bergamot	1452	1468
Pinene	1452	1468
Untreated	1454	1468
Alginate/fucoidan	1454	1468
Menthol-alginate/fucoidan	1454	1469
Linalool-alginate/fucoidan	1452	1468
Bergamot-alginate/fucoidan	**1456**	1468
Pinene-alginate/fucoidan	1452	1468

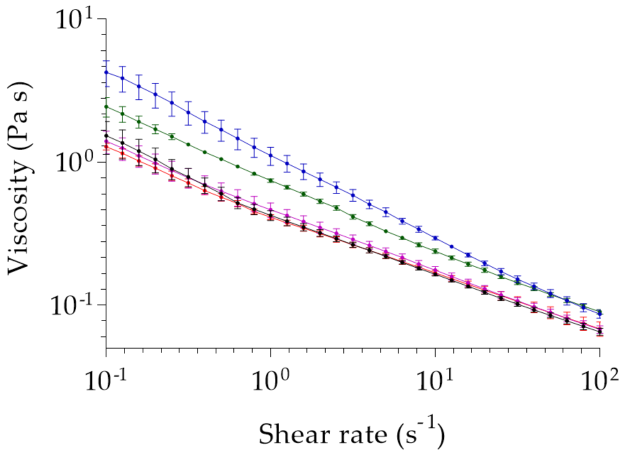

(a)

	Initial viscosity (Pa s) (0.1 s^{-1})	Final viscosity (Pa s) (100 s^{-1})
Alginate/fucoidan	1.528 ± 0.325	0.065 ± 0.003
Menthol-alginate/fucoidan	4.236 ± 0.711	0.087 ± 0.005
Linalool-alginate/fucoidan	1.396 ± 0.207	0.068 ± 0.003
Bergamot-alginate/fucoidan	1.283 ± 0.056	0.068 ± 0.006
Pinene-alginate/fucoidan	2.442 ± 0.313	0.090 ± 0.003

(b)

Figure S1. Analysis of (a) viscosity as a function of shear rate of alginate/fucoidan (black), menthol-alginate/fucoidan (blue), linalool-alginate/fucoidan (magenta), bergamot-alginate/fucoidan (red) and pinene-alginate/fucoidan (green). Calculated values of initial (minimum shear rate – 0.1 s^{-1}) and final (maximum shear rate – 100 s^{-1}) viscosity (b). Data expressed as mean ± SD of n=3 different hydrogel batches.

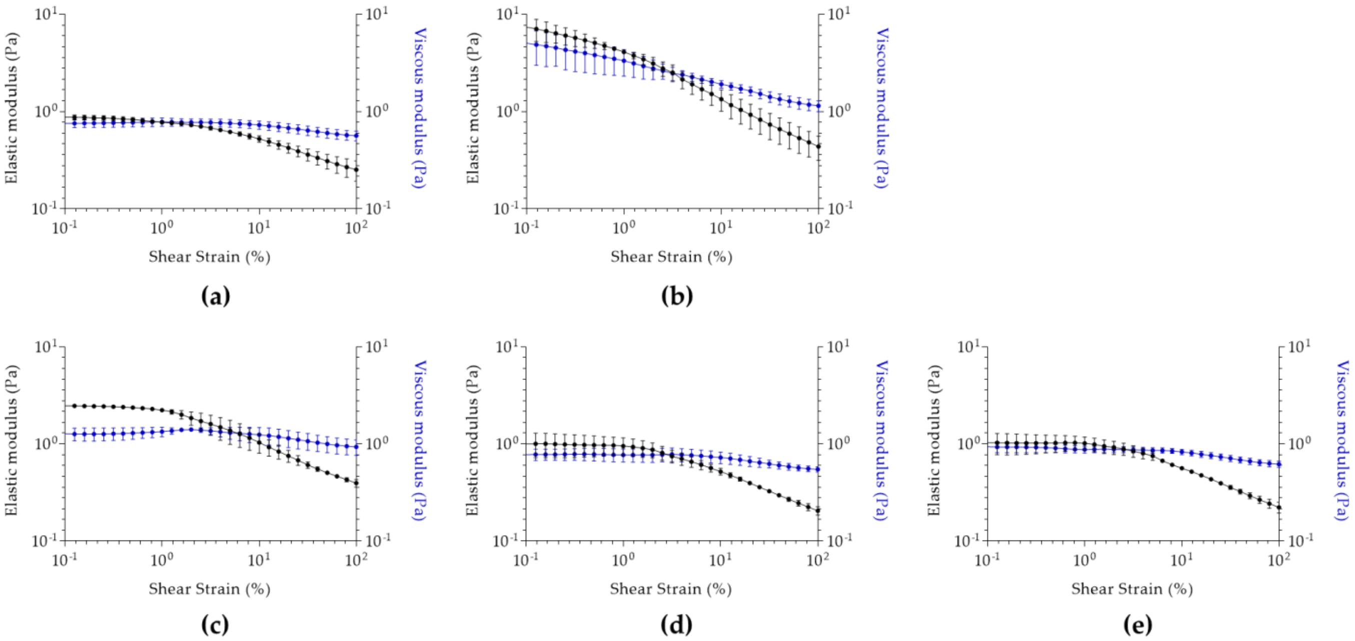

Figure S2. Resistance to deformation analysis from determination of linear viscoelastic region of (a) alginate/fucoidan, (b) menthol-alginate/fucoidan, (c) linalool-alginate/fucoidan, (d) bergamot-alginate/fucoidan and (e) pinene-alginate/fucoidan. Data expressed as mean ± SD of n=3 different hydrogel batches.

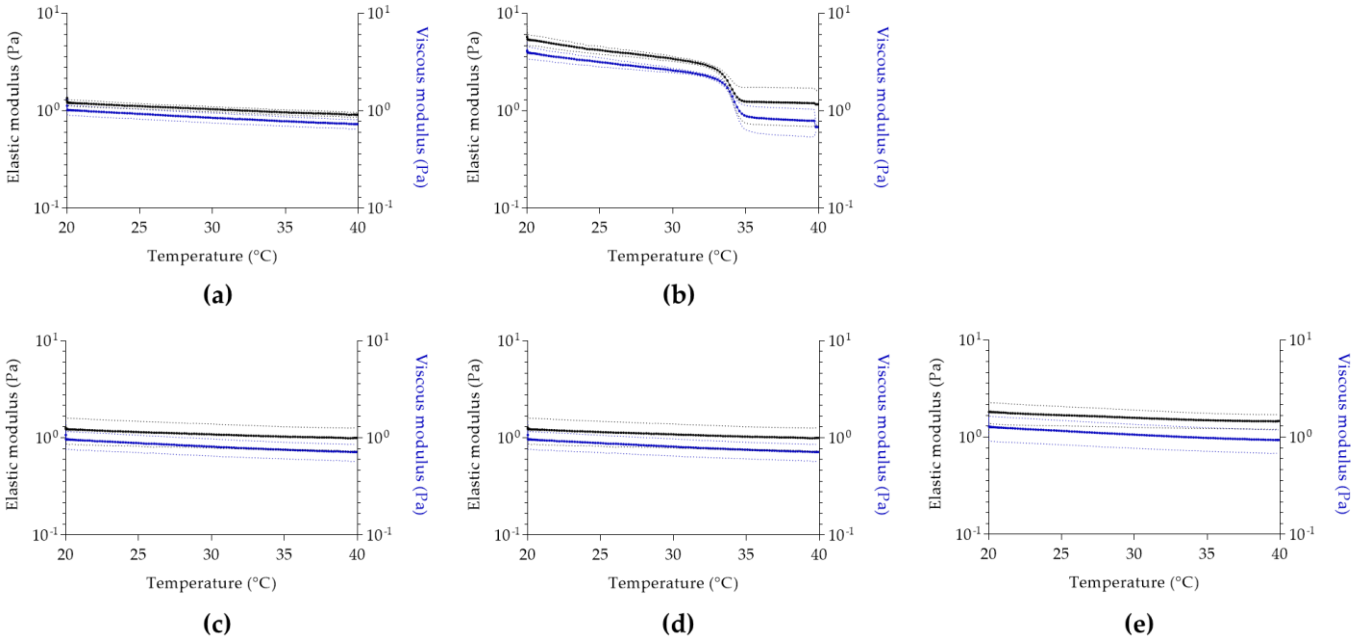

Figure S3. Resistance to temperature ramp from 20 to 40 °C of (a) alginate/fucoidan, (b) menthol-alginate/fucoidan, (c) linalool-alginate/fucoidan, (d) bergamot-alginate/fucoidan and (e) pinene-alginate/fucoidan. Data expressed as mean ± SD of n=3 different hydrogel batches.

Disclaimer/Publisher's Note: The statements, opinions and data contained in all publications are solely those of the individual author(s) and contributor(s) and not of MDPI and/or the editor(s). MDPI and/or the editor(s) disclaim responsibility for any injury to people or property resulting from any ideas, methods, instructions or products referred to in the content.
